# Systematic review of outcome measures in randomised controlled trials of pediatric eosinophilic esophagitis (EoE) treatment

**DOI:** 10.1186/1710-1492-10-S1-A70

**Published:** 2014-03-03

**Authors:** Tamar Rubin, Jacqueline Clayton, Denise Adams, Rabin Persad, Sunita Vohra

**Affiliations:** 1 Department of Pediatrics, University of Alberta, Edmonton, Alberta, Canada; 2 CARE Program, University of Alberta, Edmonton, Alberta, Canada; 3 Department of Public Health Sciences, University of Alberta, Edmonton, Alberta, Canada; 4 Department of Pediatric Gastroenterology, University of Alberta, Edmonton, Alberta, Canada

## Background

Heterogeneity has been noted in the selection and reporting of disease-specific pediatric outcomes in randomized controlled trials (RCTs) [1]. The consequence may be invalid results from RCTs, or difficulty in comparing results across trials [2,3]. The primary objective of this systematic review was to assess the heterogeneity of outcome measures selection and reporting in recent pediatric EoE treatment trials. As secondary objectives, we assessed the heterogeneity of disease definition and resolution across studies compared to established concensus guidelines, as well as the evidence for current EoE treatments.

## Methods

We searched MEDLINE, EMBASE, The Cochrane Library, Cochrane Central Register of Controlled Trials (CENTRAL), and CINAHL from the last ten years, including randomized controlled trials of EoE treatment in patients 0-18 years. Two authors independently assessed articles for inclusion.

## Results

**Figure 1:**
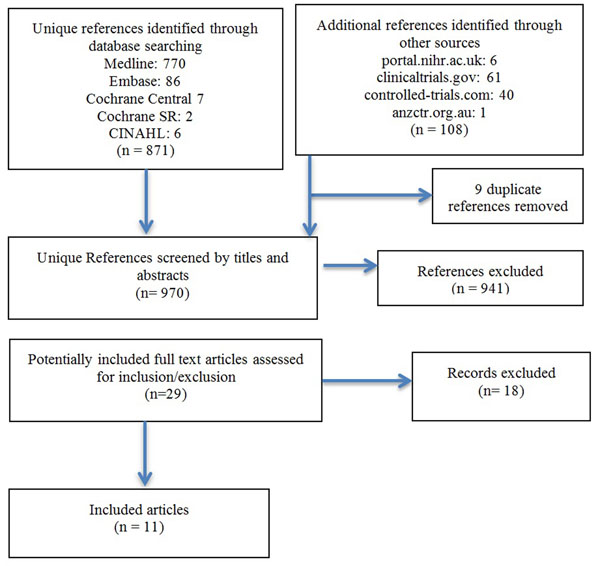
Flow Diagram

A total of 11 studies met inclusion criteria (Fig 1). Numerous outcome measures were selected and reported in these trials, with certain measures, such as esophageal eosinophilia, clinical symptoms, safety, histologic features, and endoscopic features, re-occurring frequently, but not universally. Uptake of consensus-established diagnostic criteria for EoE (FIGER criteria) was 30% in trials published after 2007. Due to the small number and heterogeneity of studies obtained, no conclusions regarding treatment efficacy could be made.

## Conclusions

The results of this study confirm the need for universally reported, pediatric-specific, standardized outcome measures in EoE trials. Adherence to standardized disease definitions will enhance the utility of outcome measures. Consistent disease definition and standardized outcome reporting will allow for meta-analyses across similar trials and thus inform future clinical decision-making in pediatric EoE.

## Systematic review registration

CRD42013003798
